# Improved SSD network for fast concealed object detection and recognition in passive terahertz security images

**DOI:** 10.1038/s41598-022-16208-0

**Published:** 2022-07-15

**Authors:** Lu Cheng, Yicai Ji, Chao Li, Xiaojun Liu, Guangyou Fang

**Affiliations:** 1 Aerospace Information Research Institute, Chinese Academy of Sciences, Beijing, 100190 China; 2 Key Laboratory of Electromagnetic Radiation and Sensing Technology, Chinese Academy of Sciences, Beijing, 100190 China; 3 School of Electronic, Electrical and Communication Engineering, University of Chinese Academy of Sciences, Beijing, 100049 China

**Keywords:** Computer science, Information technology, Scientific data, Applied optics, Optical techniques, Data processing, Image processing, Machine learning, Electrical and electronic engineering

## Abstract

With the strengthening of global anti-terrorist measures, it is increasingly important to conduct security checks in public places to detect concealed objects carried by the human body. Research in recent years has shown that deep learning is helpful for detecting concealed objects in passive terahertz images. However, previous studies have failed to achieve superior accuracy and performance for real-time labeling. Our research aims to propose a novel method for accurate and real-time detection of concealed objects in terahertz images. To reach this goal we trained and tested a promising detector based on deep residual networks using human image data collected by passive terahertz devices. Specifically, we replaced the backbone network of the SSD (Single Shot MultiBox Detector) algorithm with a more representative residual network to reduce the difficulty of network training. Aiming at the problems of repeated detection and missed detection of small targets, a feature fusion-based terahertz image target detection algorithm was proposed. Furthermore, we introduced a hybrid attention mechanism in SSD to improve the algorithm’s ability to acquire object details and location information. Finally, the Focal Loss function was introduced to improve the robustness of the model. Experimental results show that the accuracy of the SSD algorithm is improved from 95.04 to 99.92%. Compared with other current mainstream models, such as Faster RCNN, YOLO, and RetinaNet, the proposed method can maintain high detection accuracy at a faster speed. This proposed method based on SSD achieves a mean average precision of 99.92%, an F1 score of 0.98, and a prediction speed of 17 FPS on the validation subset. This proposed method based on SSD-ResNet-50 can provide a technical reference for the application and development of deep learning technology in terahertz smart security systems. In the future, it can be widely used in some public scenarios with real-time security inspection requirements.

In recent years, the global counter-terrorism situation has become increasingly severe. Security inspections in public places have gradually attracted widespread attention from various countries. In crowded public places, in addition to the security checks on the packages people carry, it is also important to conduct security checks on the human body to detect dangerous hidden objects. However, the existing security inspection systems have some deficiencies more or less. In this case, the terahertz security inspection system using electromagnetic waves as the detection method is playing an increasingly important role.

Terahertz waves refer to electromagnetic waves with frequencies between 0.1 and 10 THz, which have similar characteristics to microwaves and infrared^1^. Terahertz imaging can detect not only metallic objects, but also non-metallic contraband such as explosives, ceramic knives, drugs, and glass knives^2,3^. Terahertz waves can penetrate insulating materials such as clothing, plastics, and ceramics^4–6^, so they can be used to detect objects hidden under human clothing. Compared with microwaves, the terahertz waves have shorter wavelengths and higher imaging accuracy. Terahertz waves have much longer wavelengths compared to X-rays and visible light. Therefore, the resolution and signal-to-noise ratio of human security imaging based on terahertz systems are quite different from X-ray imaging and visible light imaging. Such characteristics make it difficult for terahertz imaging to automatically detect hazardous objects using existing object detection algorithms. Terahertz imaging systems are divided into active and passive working modes. In the application of detecting concealed objects in the human body, passive terahertz imaging systems occupy a major position^7,8^. Passive terahertz imaging systems do not emit electromagnetic waves and will not cause harm to the human body. The current speed of passive terahertz imaging is as high as 10 frames per second, which puts forward higher requirements for the speed and accuracy of detection. At present, most of the images acquired by the terahertz systems are checked manually one by one, which not only consumes a lot of manpower, but also may lead to missed inspections when people mark images for a long time. As a result, the advancement of automatic identification technology for passive terahertz images is critical for the widespread adoption of intelligent security inspection scenarios.

It is well known that the security screening of terahertz imaging systems is designed to detect and identify the threat of dangerous goods carried by the human body. More and more terahertz passive imaging systems have been studied in recent years, but theories and methods for identifying or locating dangerous objects from terahertz images are still in their infancy. Compared with optical and active terahertz imaging, passive terahertz images have a low signal-to-noise ratio and are easily affected by background variations. These characteristics make the use of passive terahertz imaging systems for security inspections a huge challenge. Traditional object detection algorithms of passive terahertz images mainly include segmentation-based detection algorithms and feature matching-based detection algorithms. The segmentation-based detection algorithm uses the grayscale information in the passive terahertz image to divide the image into two regions, the target and the background, and then detects the target. At present, the commonly used segmentation algorithms include maximum entropy segmentation method^9^, Otsu method^10^, region growing method, etc. The target segmentation-based algorithm is fast and easy to implement, but it can’t detect complex targets. The feature matching-based detection algorithms construct a target feature descriptor to match known prior feature information. Common features include Haar features^11^, gradient histograms^12^ (HOG), dense SIFT features^13^, etc. Feature-based detection methods rely on manual feature extraction. However, since artificially constructed features are usually limited to specific object types, satisfactory object detection results cannot be achieved. In recent years, with the development of deep learning, the application of deep convolutional neural networks in optical image target detection has achieved good results, and its detection accuracy is far superior to traditional methods^14,15^. Low-resolution passive terahertz images contain very little information, making it very difficult to extract features from images manually. The CNN network can automatically extract features from passive terahertz images. Although large-scale CNN networks have good detection and classification effects, they cannot meet real-time performance due to large parameters and complex structures. Extensive research shows that there is still space for improvement in the accuracy and speed of current CNN-based terahertz image target detection algorithms^16–18^. Hence, there is an urgent need to propose a method with sufficient accuracy and robustness for object recognition in passive terahertz images. Nevertheless, to the best of the author’s knowledge, there are only a handful of studies in the area.

There are two main problems in the direct use of deep learning algorithms in the field of passive terahertz image object recognition, one is that the accuracy of object detection is not high, and the other is that the speed of object detection is not fast enough. In response to these two issues, we chose the single-stage detection algorithm SSD, which has relatively high accuracy and speed in the natural light object detection algorithm, as the basic model. This is the first application of the SSD algorithm in the field of passive terahertz image object detection. We investigate the potential of the residual network ResNet-50 in terms of accuracy and real-time performance in detecting concealed objects in passive terahertz images. The method described in this paper aims at selecting the most efficient CNN architecture and further exploring the ways of its modification and optimization to ensure superior real-time classification potential for detecting concealed objects. The significance and originality of this research lie in the first exploration of the application of the SSD model in the field of passive terahertz image detection and several important improvements are proposed for image characteristics. The ultimate goal of this research is to develop a fast and accurate detection algorithm for passive terahertz images and promote its practical application in intelligent security inspection scenarios. We propose the following improvements to further improve the accuracy and speed of the algorithm in detecting concealed objects. To summarize, our main contributions are as follows:

- Aiming at the network degradation problem of the VGGNet network^19^ in the SSD algorithm, the improved algorithm uses the ResNet-50 network^20^ as the feature extraction network.
- For the problem of poor detection performance of small objects, we extract multi-level features to form a feature pyramid network^21^, which is used to detect objects of different scales. Through upsampling, deep features and shallow features are fused to construct feature representations with rich semantic information, making the fused features more descriptive.
- The proposed algorithm introduces the spatial-channel attention mechanism into the SSD network to enhance the semantic information of high-level feature maps. Therefore, the ability of the algorithm to obtain the details and position information of the object can be improved, thereby reducing the missed detection rate and false detection rate, and improving the detection accuracy of small objects.
- This paper introduces the Focal Loss^22^ to improve the imbalance of positive and negative samples and hard and easy samples. By increasing the weight of the hard samples in the loss function, the robustness of the proposed algorithm can be improved.

Compared with the unoptimized SSD algorithm, this method can improve the detection accuracy and at the same time meet the detection speed requirements in intelligent security inspection scenarios. Specifically, our proposed algorithm achieves a mean average precision of 99.92%, and the detection speed of the algorithm can reach 17 FPS, which can meet the real-time requirements. This is an excellent outcome that could not have been obtained with earlier methods. The remainder of the paper is organized as follows: The related works on terahertz image object detection and the basic introduction to SSD algorithm are introduced in “Related work”. In the following section, the proposed improved SSD algorithm is presented in detail. Section “Experimental results and discussion” shows and analyzes the experimental results of our method after its deployment. The conclusion of the paper is formed in the last section.

## Related work

This section introduces the related works, including the introduction of common object detection methods based on CNN models, the basic introduction of the SSD model, and related work on terahertz image object detection.

### Object detection and recognition based on CNN models

In recent years, deep learning has made breakthroughs in object visual detection. At present, object detection algorithms based on deep learning models are mainly divided into two categories: one is various two-stage algorithms based on R-CNN^23^, including Fast R-CNN^24^, Faster R-CNN^25^, Mask R-CNN^26^, RFCN^27^, etc. These two-stage algorithms greatly improve the detection accuracy, but the two-stage detection results in a slow detection speed, which cannot meet the real-time requirements. The other category is one-stage detection algorithms represented by YOLO^28^ (You Only Look Once) and SSD^29^. The YOLO algorithm uses the idea of regression to greatly improve the detection speed, but its recognition accuracy of object location is poor and the recall rate is low. The SSD algorithm combines the regression method in the YOLO algorithm, not only follows the anchor mechanism in Faster R-CNN but also extracts multi-scale feature maps for classification and prediction, which greatly improves the precision and speed of object detection.

### Detailed introduction of SSD algorithm

**Figure 1:**
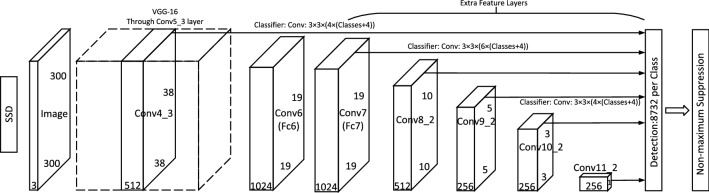
The network structure of SSD.

The SSD algorithm is an object detection algorithm proposed by Wei Liu et al.^29^, which combines the anchor mechanism in Faster R-CNN and the regression idea in YOLO. The SSD algorithm not only has a clear speed advantage over Faster R-CNN, but also has a clear accuracy advantage over YOLO. It is based on the VGGNet-16 backbone and fuses feature maps of different scales for feature extraction. Its network structure is divided into two parts: one is the feature extraction network, and the other is the classification and regression layer. Specifically, the feature extraction network can extract the feature information of the object in the image, and at the same time can improve the ability of the network to perceive the object. The classification and regression layers are capable of classifying and regressing each candidate box to detect objects in the terahertz images. The structure of the original SSD network is shown in Fig. 1.

As we all know, the SSD algorithm no longer uses fully connected layers, which improves computational efficiency. Moreover, the SSD algorithm uniformly modifies the size after the input image, and there is no mandatory requirement for the size of the input image. Besides, SSD directly uses convolution to calculate candidate boxes and predict classification in one step, which can simplify the process. However, the SSD algorithm also has some disadvantages. To begin with, the maximum size of feature maps used for prediction has a resolution of 3838. If the size of small objects in the input image is small, the detailed information contained in the lower layers can easily be lost after the pooling layer. Besides, as the feature map size of additional feature extraction networks continues to decrease, the feature extraction and representation capabilities of shallow feature layers are limited. While the feature output layer of deep features is mainly responsible for large objects, resulting in poor detection performance of SSD for small objects. Finally, the convolutional layer of SSD algorithm feature extraction cannot take into account the features of adjacent feature layers of different scales. This results in insufficient feature extraction capability for small object features in complex environments during object feature extraction. All in all, the detection performance of the original SSD algorithm for small objects in complex environments needs to be improved. To meet the need of detecting hidden objects in passive terahertz images, some improvements to its network structure are also required.

### Hidden object detection in terahertz security images

In recent years, passive terahertz imaging technology has shown significant development prospects in the field of security inspection. It is of great significance to detect dangerous objects hidden in the human body in passive terahertz security images. At present, some scholars have carried out research on passive terahertz human body security image object detection algorithms^30,31^. According to the characteristics of terahertz images, Zhang et al. adopted the Otsu threshold segmentation algorithm and applied the contour tracking method to extract contours, which can realize object detection in human terahertz images^32^. Aiming at the difficulty of detecting edge objects in human terahertz images, Jiang et al. proposed an automatic recognition algorithm for human edge objects^33^. Santiago Lopez Tapia et al. proposed a method combining image processing and statistical machine learning techniques to solve the problem of object localization detection in terahertz images^34^. Niu et al. proposed a terahertz human image processing and recognition method based on the principle of saliency and sparse coding, which can realize automatic recognition of hidden objects in the human body^35^.

In these methods, the objects were first separated from the image and then classified and recognized in a complex and slow process. Moreover, the above traditional object detection methods have certain defects and poor generalization ability. The performance of these algorithms is often affected by the complexity of the image background. The simpler the image background, the higher the object detection efficiency and the better the detection performance. On the contrary, once the image background becomes complex, the efficiency and performance of object detection will decrease accordingly.

The convolutional neural network CNN^36^ (convolutional neural network) based on deep learning technology can solve the defects of traditional methods well. CNN can not only complete feature extraction, but also has good robustness and strong feature expression ability. These properties enable it to accurately localize detection objects in both simple and complex environments. Liu Chen et al. introduced Focal Loss^22^ on the basis of Faster R-CNN and detected concealed objects in active millimeter-wave images without using transfer learning^16^. Hong Xiao et al. proposed the R-PCNN algorithm. The algorithm adds traditional image preprocessing methods to the front end, which improves the speed and accuracy of object detection and recognition^17^. Xi Yang et al. exploited the spatiotemporal information of terahertz image sequences and used CNN to achieve automatic object detection and recognition^18^. Extensive studies show that there is still space for improvement in the accuracy and speed of current CNN-based terahertz image object detection algorithms.

## Methods

**Figure 2:**
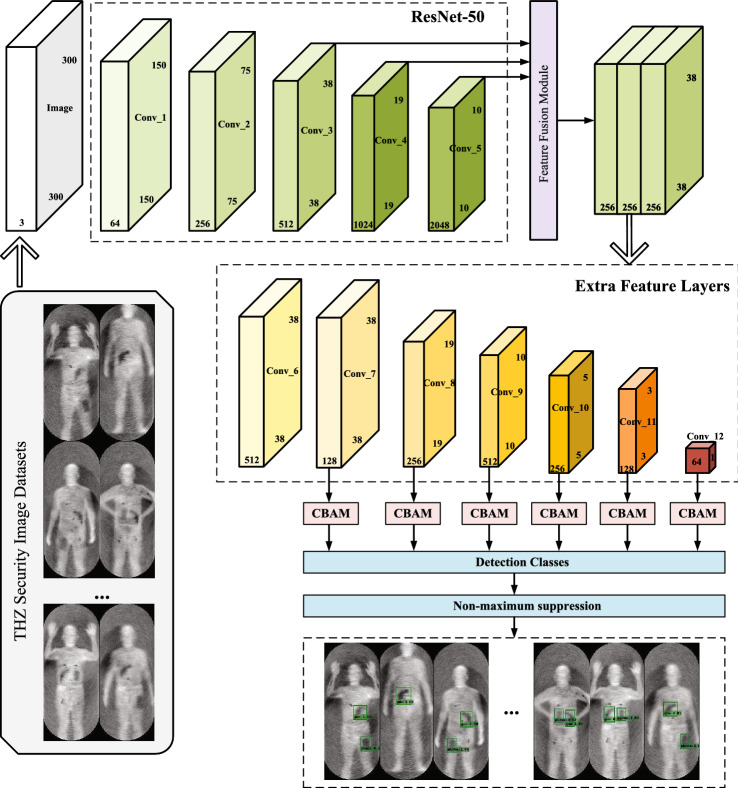
Improved SSD network architecture.

According to the characteristics of terahertz images, we propose a novel method for object detection in terahertz human security images. This method enables accurate real-time detection of concealed objects in terahertz images. The improved SSD framework is shown in Fig. 2. In general, the improved SSD object detection algorithm is divided into the following four parts. Firstly, the basic network VGGNet-16 is replaced with a deep convolutional network ResNet-50 to enhance the feature extraction ability. Additional feature layers are introduced into the basic network to further enhance the feature expression capability of the object detection layer. Afterward, three feature maps of different scales are fused in the feature extraction layer to enhance the semantic information correlation between the front and rear scale maps. In the next parts, a hybrid attention mechanism is introduced into SSD to enhance the semantic information of high-level feature maps. This method can improve the algorithm’s ability to obtain object details and position information, thereby reducing the missed detection rate and false detection rate. Finally, the Focal Loss function is introduced to improve the robustness of the algorithm by increasing the weight of negative samples and hard samples in the loss function.

**Figure 3:**
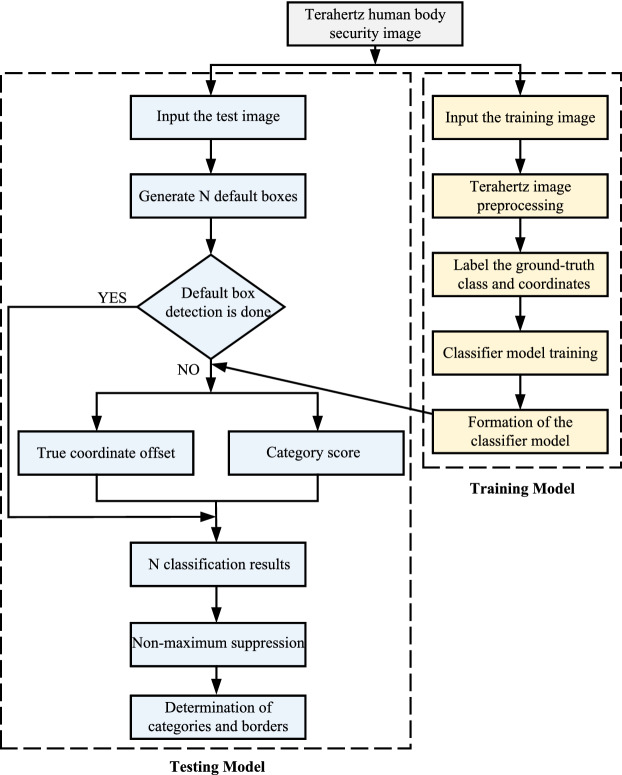
Flowchart of the algorithm.

The overall flowchart of our proposed algorithm is shown in Fig. 3. During the training phase, the objects in the input image are marked to obtain the location information and category information of the real target. Then the model is trained to generate the final improved SSD object detection model. During the testing phase, each test image generates N boxes that may contain objects. Ground truth offsets and class scores are then computed using the model generated during the training phase, resulting in N classification results per image. Finally, the non-maximum suppression algorithm is used to output the final result.

### Ethical statement

This study conforms to the ethical guidelines of the Declaration of Helsinki revised in 2013. The study was approved by the ethics committee of the Aerospace Information Research Institute, Chinese Academy of Sciences. All experiments were performed by relevant guidelines and regulations. We confirmed that informed consent had been obtained from all subjects. All images were deidentified before inclusion in this study.

### Improvement of feature extraction network

**Figure 4:**
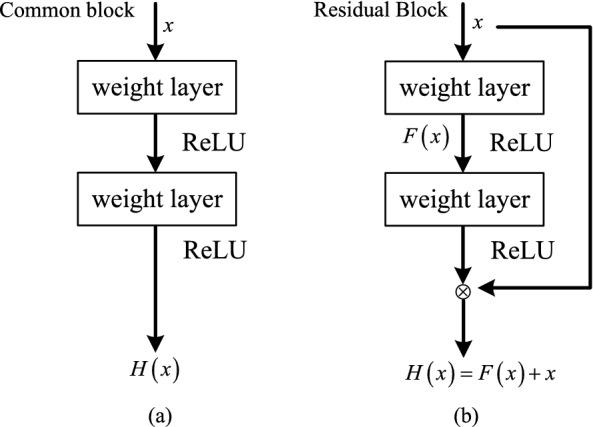
Comparison diagram of traditional structure and residual structure. (**a**) The traditional structure. (**b**) The residual structure.

The VGGNet-16 network is a simple stack of traditional CNN structures with many parameters. As the number of network layers deepens, there are not only the phenomenon of gradient disappearance and gradient explosion, but also the problem of network degradation. In response to the problems existing in the traditional CNN structure, the team of Kaiming He of MSRA proposed a convolutional neural network called Deep Residual Network(ResNet)^20^, which successfully solved the above two problems by introducing a batch normalization (BN) layer and residual block. The BN layer normalizes the feature maps of the middle layer to speed up the network convergence and improve the accuracy. The residual block fits the residual map by adding a skip connection layer between the network layers. It can directly transfer the optimal weight of the front layer to the back layer, so as to achieve the effect of eliminating network degradation. The comparison between the ordinary structure and the residual structure is shown in Fig. 4. The ResNet network adopts the jump structure in Fig. 4b as the basic network structure, also known as Bottleneck. The jump structure enables the ResNet network to have deeper layers and relatively better network performance than ordinary networks. As shown in Fig. 4, *H*(*x*) is called the desired map for stacking several network layers, *x* is the entry of the current stacking block, and the use of the ReLU activation function shortens the learning cycle. Assuming that *n* nonlinear layers can be approximately expressed as a residual function, then the stacked network layers are fitted to another map $F(x)=H(x)-x$, and the final base map becomes $H(x)=F(x)+x$. The idea of residual structure can effectively solve the problem of network performance degradation and gradient, and also improve network performance. By constructing residual learning, the residual network can approximate a better-desired mapping by approximating the coefficients of multiple nonlinear connections to zero.

The detection of concealed objects in passive terahertz images is affected by complex backgrounds and similar interference. Research has proved that deepening the network structure is effective for improving feature detection. However, VGGNet-16 is a relatively shallow network, which is insufficient to extract features of hidden objects. Therefore, we replace the feature extraction network with ResNet-50, a network with deeper and richer semantic information. ResNet-50 has a total of 16 Bottlenecks, each of which contains 3 convolutional layers. Together with the input layer and the final fully connected layer, a 50-layer residual network is formed. The comparison of network parameters and complexity between VGGNet-16 and ResNet-50 is shown in Table 1. It can be seen from Table 1 that the parameters and floating-point operations of the ResNet-50 network are much lower than those of the VGGNet-16, which proves that ResNet-50 is lighter and has the conditions for faster speed.

**Table 1:** Comparison of network complexity between VGGNet-16 and ResNet-50.

Network	Parameter quantity	Number of floating-point operations
VGGNet-16	138	15.5
ResNet-50	25.5	3.8

Compared to AexNet, GoogleNet, and VGGNet-16, ResNet-50 is known for its ability and performance to achieve lower error rates and higher accuracy output than other architectures^37^. The depth of each convolutional layer of ResNet-50 is higher than that of VGGNet-16, which enhances the network learning ability and further improves the feature extraction ability of concealed objects in images. ResNet-50 introduces a residual module in the convolutional layer, which solves the problem of training degradation caused by the deepening of the network. The network has high convolution calculation efficiency and reduces the redundancy of the algorithm. Taking into account factors such as accuracy and detection speed, the novel CNN network based on the ResNet-50 network is more accurate than VGGNet-16. By using ResNet-50 as the backbone network, the data features can be better extracted for classification and loss computation. The improved algorithm uses ResNet-50 as the extraction network, removes the fully connected layer in ResNet-50, adds several additional convolutional layers to extract features, and obtains feature maps of different scales. In this way, the limitation of the fully connected layer on the size of the input image can be solved. The network can obtain feature maps of different sizes to construct the detection part of the feature maps of different sizes in the network. The size of the feature map of the feature extraction layer of the improved algorithm is 3838, 1919, 1010, 55, 33 and 11, and the dimensions are 128, 256, 512, 256, 128 and 64 respectively. The shallow convolution layers extract relatively small features, while deep convolution layers can extract richer information. Therefore, shallow convolutional feature maps and deep convolutional feature maps are used to detect small and large objects, respectively. Replacing the original front-end network VGGNet-16 with the ResNet-50 network can improve the accuracy on the basis of the original SSD algorithm, but there are still problems such as poor real-time performance, false detection, and repeated detection of small objects. Therefore, feature fusion techniques are used to improve the SSD algorithm.

### Feature fusion module

The SSD algorithm takes full advantage of multiple convolutional layers to detect objects. It has better robustness to the scale changes of objects, but the disadvantage is that it is easy to miss small objects. The network structure of the SSD algorithm is shown in Fig. 1. Each convolutional layer corresponds to the scale of an object, thus ignoring the relationship between layers. The low-level feature layer Conv_3 has a resolution size of 3838 and contains a lot of edges and non-target information. Conv_4 and Conv_5 have higher resolution and basic contour information and thus have richer and more detailed feature representation. As the number and depth of network layers increase, the scale of convolutional layers gradually decreases, and the semantic information becomes more and more abundant. However, the underlying conv4_3 does not utilize high-level semantic information, resulting in poor detection of small objects.

**Figure 5:**
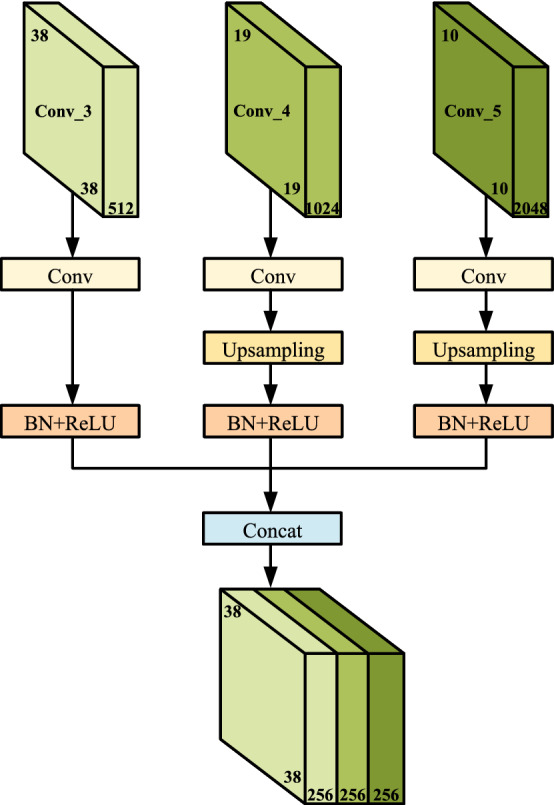
Overview of the proposed feature fusion strategy.

The improved algorithm fuses adjacent low-level high-resolution feature maps with clear and detailed features and high-level low-resolution feature maps with rich semantic information. Feature fusion can enhance the ability of the low-level feature layer to fully express the detailed features of small objects, and improve the detection effect of the SSD algorithm on small objects. The feature fusion structure proposed in this paper is shown in Fig.  5. The improved SSD model performs upsampling operations on Conv_4 and Conv_5 by bilinear interpolation, and performs feature fusion with Conv_3. Better upsampling results can be obtained by using bilinear interpolation operations instead of adjacent interpolation operations. We use the concatenation method to fuse the high-semantic feature maps of Conv_4 and Conv_5, and use them to supplement the feature information of the convolutional feature maps of the Conv_3. Moreover, we still use the scale of Conv_3 as the scale of the new prediction stage. This way of passing down the fusion information can further fuse high-semantic information in deep layers and detailed information in shallow layers.

As we all know, the update of the parameters of the previous convolutional layer will cause the data distribution of the later input layer to change, resulting in a large difference in the data distribution of the convolutional feature layer. Therefore, there will be large differences between feature maps, and feature connection operations cannot be performed directly. When the network layer changes slightly, the features are accumulated and amplified through the fusion layer, resulting in slow convergence of the algorithm. Therefore, a BN layer is added after each feature layer for normalization. BN layers can reduce the effects of a slow training process due to increased model complexity. The improved SSD algorithm adopts the method of multi-level fusion of different scales, combined with the idea of FPN^21^, and transfers the feature information of feature maps of different scales from top to bottom. Feature fusion can provide feature representations with rich semantic information, improving the descriptiveness of fused features.

### Attention mechanism CBAM

**Figure 6:**
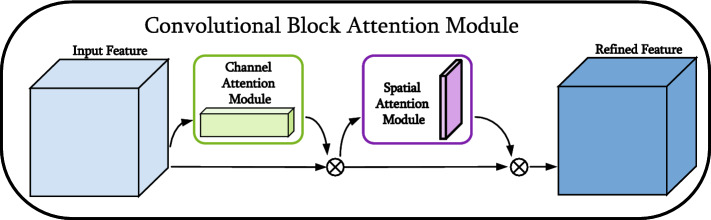
The overview of CBAM attention mechanism module.

The feature maps extracted by the feature extraction network not only have object features but also have similar interference features. The concealed objects on the human terahertz image and other similar objects are given the same importance on the feature maps, which is not conducive to the detection of hidden objects in complex backgrounds. Therefore, to improve the object recognition ability of feature maps for specific regions and specific channels, and reduce the negative interference of complex backgrounds and similar objects, the attention mechanism is applied in both channel and space dimensions. Mechanism of Attention CBAM^38^(Convolutional Block Attention Module) is a little universal module that saves computational resources and parameters. For a given feature map, attention weights are sequentially derived along both spatial and channel dimensions, and then the features are adaptively adjusted by multiplying with the original feature map. The implementation of the attention mechanism includes two modules: channel attention and spatial attention. The structure of the CBAM attention mechanism module is shown in Fig. 6. Usually, the two modules of channel attention and spatial attention are combined in turn, and better results can be achieved by placing the channel attention in the front. The improved algorithm connects the CBAM dual attention mechanism after each output feature map to improve the network’s attention to concealed objects.

**Figure 7:**
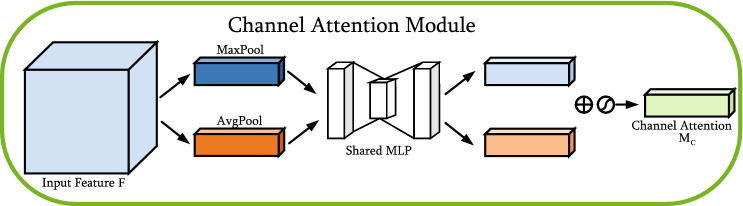
Diagram of channel attention module.

**Figure 8:**
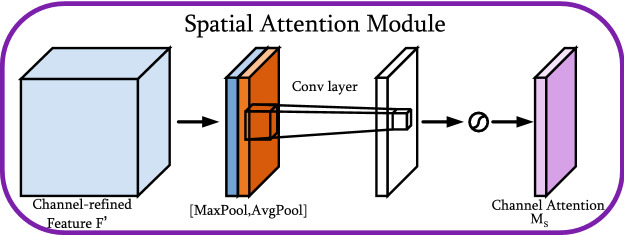
Diagram of spatial attention module.

Given an intermediate feature map $F \in \mathbb {R}^{C \times H \times W}$as input, CBAM sequentially infers a 1D channel attention map $M_{r} \in \mathbb {R}^{C \times 1 \times 1}$ and a 2D spatial attention map $M_{s} \in \mathbb {R}^{1 \times H \times W}$ as shown in Fig. 6. The overall attention progression can be summarized as:

1 $$\begin{aligned}&F^{\prime }=M_{c}(F) \otimes F \end{aligned}$$

2 $$\begin{aligned}&F^{\prime \prime }=M_{s}\left( F^{\prime }\right) \otimes F^{\prime } \end{aligned}$$

where $\otimes$ represents for element-wise multiplication. During multiplication, the attention values are broadcast accordingly: channel attention values are broadcast along the spatial dimension and vice versa. $F^{\prime \prime }$ is the final refined output. Figures 7 and 8 describe the computation process of each attention map. The details of each attention module are described below.

#### Channel attention module

The structure of the channel attention module is shown in Fig. 7. The channel attention module aggregates spatial information of feature maps by using average pooling and max pooling operations, generating two different spatial context descriptors: $F_{a v g}^{c}$ and $F_{max}^{c}$, which denote average-pooled features and max-pooled features respectively. The two descriptors are then forwarded to the shared network to generate the channel attention map $M_{c} \in \mathbb {R}^{C \times 1 \times 1}$. The shared network consists of a multilayer perceptron (MLP) with one hidden layer. To reduce parameter overhead, the hidden activation size is set to $\mathbb {R}^{C / r \times 1 \times 1}$, where *r* is the reduction ratio. After applying the shared network to each descriptor, the channel attention module merges the output feature vectors using element-wise summation. Briefly, the channel attention module is computed as:

3 $$\begin{aligned} \begin{aligned} M_{c}(F)&=\sigma ({\text {MLP}}({\text {AvgPool}}(F))+{\text {MLP}}({\text {MaxPool}}(F))) \\&=\sigma \left( W_{1}\left( W_{0}\left( F_{a v g}^{c}\right) \right) +W_{1}\left( W_{0}\left( F_{\max }^{c}\right) \right) \right) \end{aligned} \end{aligned}$$

where $\sigma$ represents the sigmoid function, $W_{0} \in \mathbb {R}^{C / r \times C}$ , and $W_{1} \in \mathbb {R}^{C \times C/r}$ . Note that both inputs share the MLP weights $W_{0}$ and $W_{1}$ ,and the ReLU activation function is followed by $W_{0}$.

#### Spatial attention module

The spatial attention module utilizes the spatial relationship of features to generate a spatial attention map. Figure 8 depicts the computation progress of the spatial attention map. Unlike channel attention, spatial attention focuses on ‘where’ is an informative part, which is complementary to channel attention. To compute the spatial attention, average pooling and max pooling operations are first applied along the channel axis and then concatenated to generate an efficient feature descriptor. On the concatenated feature descriptor, a convolution layer is applied to generate a spatial attention map $M_{s}(F) \in \mathbb {R}^{H \times W}$, which encodes locations to be emphasized or suppressed. The specific operation progress is as follows.

The channel information of the feature maps is aggregated using two pooling operations to generate 2D maps: $F_{a v g}^{s} \in \mathbb {R}^{1 \times H \times W}$ and $F_{max}^{s} \in \mathbb {R}^{1 \times H \times W}$. Each represents the average-pooled and max-pooled features across the channel. They are then concatenated and convolved through standard convolutional layers to generate a 2D spatial attention map. To sum up, the spatial attention is calculated as:

4 $$\begin{aligned} \begin{aligned} M_{s}(F)&=\sigma \left( f^{7 \times 7}([{\text {AvgPool}}(F) ; {\text {MaxPool}}(F)])\right) \\&=\sigma \left( f^{7 \times 7}\left( \left[ F_{\text{ ang } }^{s} ; F_{\max }^{s}\right] \right) \right) \end{aligned} \end{aligned}$$

where $\sigma$ represents the sigmoid function and $f^{7 \times 7}$ denotes a convolution operation with a filter size of 77.

The improved algorithm is based on SSD-ResNet-50 and adds CBAM modules after 6 feature maps of different sizes. By adjusting the size of the feature maps, each feature map can remain the same size as before the output after the feature weighting of the CBAM module. The attention mechanism further enhances the semantic information of high-level feature maps, reduces the target missed detection rate, and improves the robustness of the algorithm.

### Improvement of the loss function

The loss function of the original SSD network consists of a weighted sum of the confidence loss and the position loss. The specific expression is calculated as:

5 $$\begin{aligned} L(x, c, l, g)=\frac{1}{N}\left( L_{c o n f}(x, c)+\alpha L_{l o c}(x, l, g)\right) \end{aligned}$$

where $L_{c o n f}$ and $L_{loc}$ denote the confidence loss and localization loss, respectively. $\alpha$ represents the weight of the localization loss, *c* and *l* are the category confidence and position offset information of the prediction box, respectively. *x* is the matching result between the previous frame and different categories, if it matches, the result is $x=1$, otherwise, it is $x=0$. Furthermore, *g* represents the offset between the ground-truth bounding box and the prior bounding boxes, and *N* denotes the number of the prior bounding boxes.

The problem of sample imbalance can be solved by adjusting the positive and negative sample ratio parameters in the input network. To solve the problem of sample imbalance in the SSD algorithm, we use the Focal Loss function^22^ to replace the confidence loss function in the original loss function. Its specific expression can be summarized as:

6 $$\begin{aligned} F L\left( p_{t}\right) =-a_{t}\left( 1-p_{t}\right) ^{\gamma } \log \left( p_{t}\right) \end{aligned}$$

where $p_{t}$ represents the classification probabilities of the different classes, and the value of $\gamma$ is greater than zero, which is used to adjust the rate at which the weights of the samples are easily divided. $a_{t}$ is a decimal between 0 and 1, which is used as a weight to adjust the proportion of positive and negative samples. For simple samples, $p_{t}$ will be relatively large, and the weight will naturally be small. On the contrary, for difficult samples, $p_{t}$ will be relatively small, and the weight will naturally be relatively large so that the network tends to use such difficult samples to update parameters.

**Figure 9:**
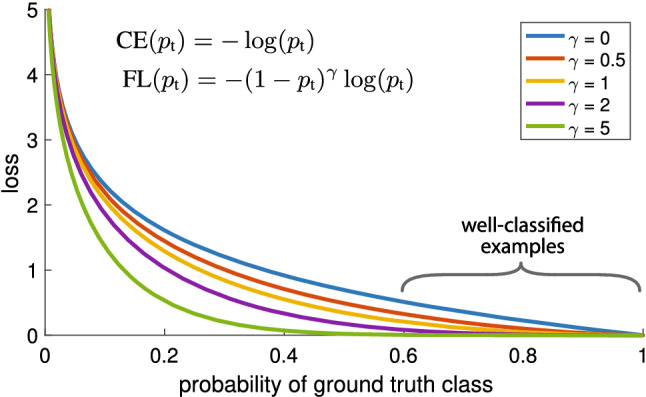
Curve of focal loss function^22^.

The Focal Loss is visualized for several values of $\gamma \in [0,5]$ in Fig. 9. The function curve is the loss function curve in SSD when $\gamma$=0. It can be seen that even the loss function value of the easily separable samples is high, resulting in a high proportion of the loss value of the easily separable samples in the algorithm. The weight of the hard samples in the input samples increases with the increase of $\gamma$, indicating that Focal Loss achieves the balance of the positive and negative samples, hard and easy samples through $a_{t}$ and $\gamma$, respectively. In this way, the samples participating in the training can be distributed more evenly, thereby further improving the reliability of the detection algorithm. In this paper, we set the parameters $a_{t}=0.25$ and $\gamma =2$ during the training process, because extensive experiments show that such parameters can achieve the best experimental results.

## Experimental results and discussion

In this section, the image dataset is introduced first, and then the evaluation metrics are described. The results of the ablation experiments demonstrate the effectiveness of the improved points proposed by this method. Extensive comparison experiments between the improved SSD algorithm and other methods are conducted in succession. In addition, we give an intuitive discussion about the visual detection effect of this algorithm and depict the ROC curve.

### Datasets

**Figure 10:**
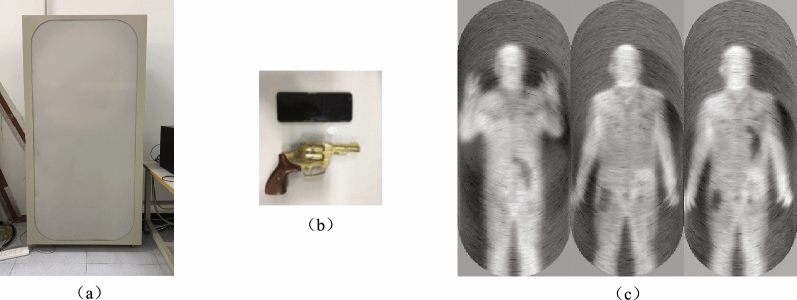
Passive terahertz hidden object imaging. (**a**) The 0.2 THz passive terahertz imaging device. (**b**) the metal pistol model and the mobile phone model. (**c**) the passive terahertz imaging results.

In the research field of terahertz concealed object detection, few passive image datasets are currently publicly available. The passive terahertz dataset we used in this paper was constructed by a team led by researcher Chao Li from the Aerospace Information Research Institute, Chinese Academy of Sciences. The dataset was collected by the 0.2 THz passive imaging device shown in Fig. 10a, a total of 896 images were collected. The size of each image is 160392, and 0 2 objects to be detected (“pistol model” and “mobile phone model”) are randomly hidden under clothes in different positions of the human torso. To ensure the authenticity and reliability of the results, the imaging experiments were carried out in the form of real people carrying concealed objects. The pistol model and mobile phone model carried by the volunteers are shown in Fig. 10b, and the imaging results are shown in Fig. 10c. In the future, we plan to gradually increase the types of objects. In addition, 896 images are expanded to 1792 images by horizontal flipping through the data augmentation method to increase the diversity of samples. The following training experiments, evaluation experiments, and comparison experiments are performed on this dataset.

### Evaluation metrics

Since passive terahertz image object detection in the security field is still a relatively “niche” field in computer vision, there is no standard evaluation protocol defined. We evaluate our proposed improved SSD algorithm using mean precision (mAP), F1 score, ROC curve, AUC value, and FPS. The following is a comprehensive overview of the indicators.

Object detection performance can be evaluated with Average Precision (AP) and mean Average Precision (mAP) between ground truth and predicted bounding box (IOU). AP is defined as the area enclosed by the precision and recall curves and the axes. The precision rate is the accuracy rate, which measures the classification ability of the object detection algorithm to detect objects. The higher the accuracy rate, the stronger the model’s ability to classify the object. The recall rate measures the detection ability of the model to the object. The higher the recall rate, the stronger the model’s ability to distinguish the object. In the object detection algorithm, the precision-recall curve (PR curve) of each category can be obtained. The area enclosed by the curve and the abscissa axis is the average precision AP value. The mAP can be obtained by averaging the APs of all classes. Under the same confidence, the larger the mAP of the model, the better the detection performance of the model. The precision and recall rate are calculated as follows:

7 $$\begin{aligned}&Precision=\frac{T P}{T P+F P} \end{aligned}$$

8 $$\begin{aligned}&Recall=\frac{T P}{T P+F N} \end{aligned}$$

**Figure 11:**
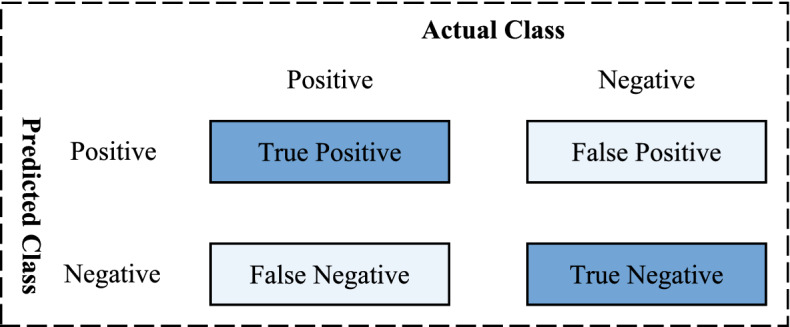
Definitions of true positive, false positive, false negative and true negative^39^. For example, “True Positive” means that one actual positive instance was predicted to be positive by one method.

True positive (TP) means that the model predicts a positive sample and the actual prediction is correct; false positive (FP) means that the model predicts a positive sample and detects it incorrectly, and false negative (FN) means that the model predicts a negative sample and detects it incorrectly. These evaluation metrics are illustrated in Fig. 11.

Additionally, the F1 score is a composite metric that combines precision and recall. The advantage of the F1 score is that it provides a single measure of quality that is easier to understand. Its calculation formula is as follows:

9 $$\begin{aligned} F1 \; Score = 2 \times \frac{Precision \times Recall}{Precision + Recall} \end{aligned}$$

A receiver operating characteristic (ROC) curve^40^, commonly used to visualize the discriminant ability of a binary classifier, is a plot of the true positive rate (TPR, sensitivity, recall) versus the false positive rate (FPR, 1-specificity) at various given cut points. A typical ROC curve is a plot of FPR on the x-axis versus TPR on the y-axis. The area under the curve (AUC) maps the entire ROC curve into a single number that reflects the overall performance of the classifier over all thresholds. Typically, the value of AUC is between 0.5 and 1. The larger the value, the better the discriminative ability of the classifier. The formulas for calculating TPR and FPR are as follows:

10 $$\begin{aligned}&TPR=\frac{T P}{T P+F N} \end{aligned}$$

11 $$\begin{aligned}&FPR=\frac{F P}{F P+T N} \end{aligned}$$

The detection speed is mainly used to evaluate the real-time performance of the algorithm. If the inference speed cannot meet the real-time requirements, our model cannot be used in the real environment. FPS (frames per second) is currently a common metric for evaluating model speed. In two-dimensional image detection, it is defined as the number of pictures that can be processed per second. The larger the value, the shorter the detection time and the faster the speed.

### Experimental details

The experimental environment of this paper is: in terms of hardware, we adopt the Inter(R)Core(TM)i5-10400F CPU processor and the graphics card of GeForce RTX 3090 Ti. In terms of software, we choose the Ubuntu 20.04 operating system, Pytorch deep learning framework, and Python 3.6 programming language.

**Figure 12:**
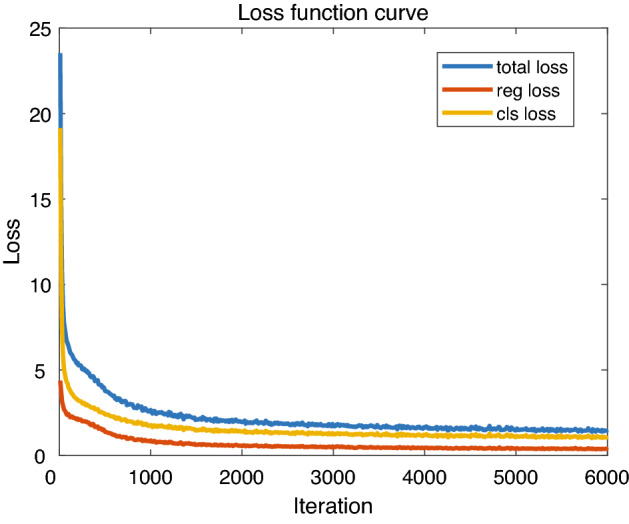
Change of loss function.

Batch_size is the number of images sent to training at one time. A larger batch_size can make model training more stable, but it will increase the amount of computation. Considering the computing power of the graphics card we have, we chose 16 as the batch_size. The learning rate is the speed at which the model is iteratively trained, and setting the learning rate correctly can make the loss curve smoother. The initial learning rate was set to 2e-4 and the learning rate decay weight was 0.001. The number of training iterations is 6000, the parameter update method is the gradient descent method with momentum (momentum SGD), and the momentum factor is set to 0.9. The resulting model was saved after every 500 iterations in the training process. After training, the object detection accuracy is used as the criterion for selecting the optimal model. The model is validated using the test set, and the experimental results are analyzed. The change of the loss function during the training process is shown in Figure 12. After 6000 iterations, the network converges. After 6000 iterations, the total loss of the model can converge to 1.62.

### Ablation study of the improved SSD algorithm

This section carries out the ablation study of the improved SSD algorithm to prove its superiority over the baseline model. We analyze the impact of improving feature extraction network, adding feature fusion network, adding attention mechanism, and improving loss function on recognition accuracy. The results of the ablation experiments are shown in Table 2.

**Table 2:** Ablation experiment results on the passive terahertz dataset.

NET	Module					mAP(%)
	VGGNet-16	ResNet-50	Feature Fusion	CBAM	Focal Loss	
SSD	$\surd$					95.04
Model-1		$\surd$				98.65
Model-2		$\surd$	$\surd$			98.77
Model-3		$\surd$		$\surd$		98.24
Model-4		$\surd$			$\surd$	95.37
Model-5		$\surd$	$\surd$	$\surd$		98.86
SSD(ours)		$\surd$	$\surd$	$\surd$	$\surd$	99.92

Model-1 replaces the backbone network from VGGNet-16 to ResNet-50, and the mAP is 3.61$\%$ higher than that of SSD. Model-2 adds a feature fusion module on the basis of adding ResNet-50, and constructs a feature with rich semantic information, indicating that mAP is 3.73$\%$ higher than SSD and 0.12$\%$ higher than Model-1. Model-3 adds a space-channel attention mechanism CBAM based on adding ResNet-50, and the mAP is 3.20$\%$ higher than that of SSD. Model-4 replaces the loss function with Focal Loss based on adding ResNet-50, and mAP is 0.33$\%$ higher than SSD. Model-5 replaces the feature extraction network with ResNet-50 on the basis of the original SSD algorithm and adds a feature fusion module and a hybrid attention mechanism CBAM. The results show that the mAP of Model-5 is 3.82$\%$ higher than that of SSD. Finally, the mAP of the fully improved SSD algorithm can reach 99.92$\%$. In summary, adding different modules significantly improves the performance of the algorithm, and these gains add up to validate the effectiveness of our algorithm.

**Figure 13:**
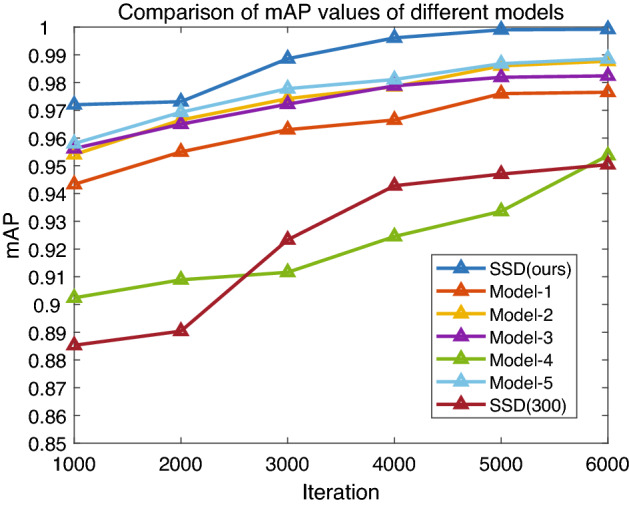
Comparision of mAP values of different models.

Furthermore, the comparison of mAP values obtained by different models in the first 6000 iterations is shown in Fig. 13. It can be seen that our proposed improved model has the highest mAP value with a high mAP of 99.92% at the 6000th iteration.

### Comparison with the state-of-the-art detection methods

We evaluated the improved SSD algorithm to other state-of-the-art detection approaches, including Faster RCNN, YOLO v5, RetinaNet, and the baseline model SSD, to verify its comprehensive performance. Table 3 shows that the improved SSD model has a detection accuracy of 99.92%. It has the best accuracy, even outperforming the existing YOLO v5 detection model. Although the improved SSD model is slower than the fastest YOLO v5, it offers a 1.56% accuracy gain. At the same time, the improved model has a speed of 17 frames per second, which is sufficient for real-time detection. The aforementioned findings suggest that our proposed enhanced algorithm outperforms other techniques.

As shown in Table 3, the detection accuracy of the improved algorithm is improved compared with the mainstream algorithms. Meanwhile, due to the increased complexity of the network, the algorithm in this paper sacrifices the speed, but it can still meet the needs of real-time detection. We believe there are two primary reasons for such superiority. (1) The baseline model of the improved SSD algorithm is suitable for this task. Its detection accuracy is only 2.71% lower than Faster RCNN, but the speed is 2.55 times faster. (2) The improved SSD algorithm can robustly improve the accuracy of the baseline model. The specific reasons can be seen in “Ablation study of the improved SSD algorithm”.

**Figure 14:**
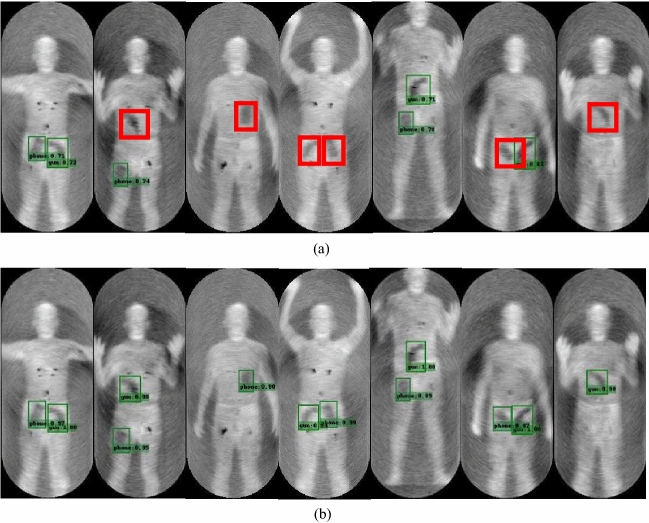
Visualized results of the passive terahertz dataset. (**a**) Detection results of the original SSD algorithm. (**b**) Detection results of the improved SSD algorithm.

We employed the passive terahertz human security image dataset given in the “Datasets” section above for detection experiments, and the visual detection results are shown in Fig. 14. As demonstrated in the red boxes in Fig. 14a, the original SSD algorithm has missed detection, and the detection confidence also has to be enhanced. The improved SSD algorithm effectively addresses the issue of missed detection while also enhancing detection confidence. The improved algorithm’s detection results are displayed in Fig. 14b. To summarize, the improved SSD algorithm outperforms the original algorithm in terms of robustness and accuracy, as well as overall performance.

**Table 3:** Comparison of accuracy with other advanced algorithms.

Model	AP(%)		mAP(%)	F1 score	Time(ms)	FPS
	Gun	Phone				
Faster R-CNN	98.30	97.20	97.75	0.95	9	4.5568
YOLO v5	99.24	97.48	98.36	0.96	42	12.2265
Retinanet	96.47	93.64	95.06	0.94	25	10.1952
SSD	96.10	93.98	95.04	0.93	23	11.2652
SSD(our)	99.94	99.90	99.92	0.98	17	8.2624

### ROC curve

**Figure 15:**
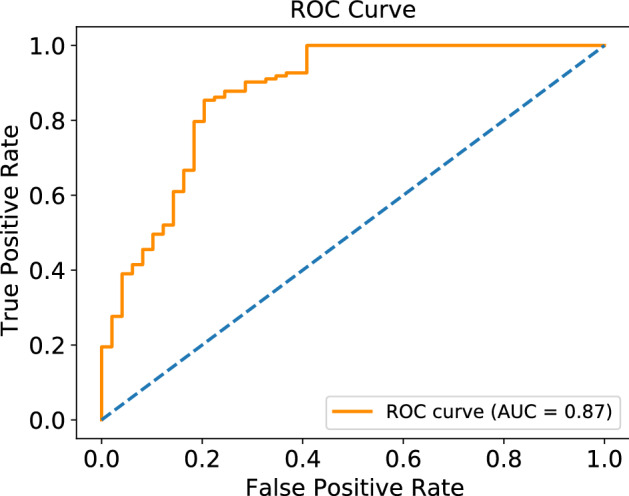
ROC curve.

The ROC curve of the proposed model is shown in Fig. 15, demonstrating the excellent recognition ability of our model for concealed objects in passive terahertz images. AUC provides summary measures for the performance of a system. It provides meaningful performance analysis. The AUC value of our proposed model is 0.87, indicating that the model has a good discriminative ability.

## Conclusion

To further improve the detection accuracy and speed of concealed objects in terahertz human security images, we proposed an improved SSD algorithm to promote its algorithm performance in this paper. First, the ResNet-50 network was used to replace the original VGGNet-16 network to overcome the VGG network degradation problem. Afterward, we designed a feature fusion module to fuse deep features and shallow features to construct features rich in semantic information, which is beneficial to the detection of small objects. In the next parts, the hybrid attention mechanism was introduced in the SSD network to improve the network’s attention to concealed objects. Finally, we introduced the Focal Loss function in the loss function to improve the robustness of the algorithm. The results of ablation experiments illustrate the efficacy of the changes described in this paper. At the same time, we also compare the proposed model with other mainstream detection methods on the terahertz human security image dataset. The results showed that our method achieves significantly improved detection accuracy in comparison with the original algorithm when the speed is only slightly reduced. The detection accuracy of the proposed method is as high as 99.9%, and the detection speed is about 17 FPS. Therefore, it can meet the real-time detection needs of security inspection scenarios.

Our approach can be improved in the future as follows: (1) The training dataset can be augmented to improve the generalization ability of the model. (2) The model can be further lightweight to reduce the amount of calculation and improve the detection speed. (3) More emphasis should be placed on the model’s interpretability in order to make the model easier to interpret and understand. It also makes the black box of the CNN-based model more transparent and reasonable. We believe that the application of Explainable AI will help to reduce the uncertainty of deep learning models. The insights gained from this research facilitate the application of deep learning techniques in smart security screening scenarios. It is believed that the method proposed in this paper will have important application value in the terahertz intelligent security systems.

## Data Availability

The datasets generated during the current study are not publicly available to non-cooperative units in the short term due to the dataset has been collected for a long time and the data involves personal privacy. However, the dataset is available from the corresponding author upon reasonable request.
